# Overdiagnosis of breast cancer in the Norwegian Breast Cancer Screening Program estimated by the Norwegian Women and Cancer cohort study

**DOI:** 10.1186/1471-2407-13-614

**Published:** 2013-12-30

**Authors:** Eiliv Lund, Nicolle Mode, Marit Waaseth, Jean-Christophe Thalabard

**Affiliations:** 1 Department of Community Medicine, Faculty of Health Sciences, University of Tromsø - The Arctic University of Norway, Tromsø, Norway; 2 Department of Pharmacy, University of Tromsø - The Arctic University of Norway, Tromsø, Norway; 3 Applied Mathematics Lab, Paris Descartes University, Sorbonne Paris Cité, Assistance Publique- Hôpitaux de Paris, UMR CNRS 8145, Paris, France

**Keywords:** Breast cancer, Mammography screening, Overdiagnosis, Hormone therapy

## Abstract

**Background:**

There is increasing ambiguity towards national mammographic screening programs due to varying publicized estimates of overdiagnosis, i.e., breast cancer that would not have been diagnosed in the women’s lifetime outside screening. This analysis compares the cumulative incidence of breast cancer in screened and unscreened women in Norway from the start of the fully implemented Norwegian Breast Cancer Screening Program (NBCSP) in 2005.

**Methods:**

Subjects were 53 363 women in the Norwegian Women and Cancer (NOWAC) study, aged 52–79 years, with follow-up through 2010. Mammogram and breast cancer risk factor information were taken from the most recent questionnaire (2002–07) before the start of individual follow-up. The analysis differentiated screening into incidence (52–69 years) and post screening (70–79 years). Relative risks (RR) were estimated by Poisson regression.

**Results:**

The analysis failed to detect a significantly increased cumulative incidence rate in screened versus other women 52–79 years. RR of breast cancer among women outside the NBCSP, the “control group”, was non-significantly reduced by 7% (RR = 0∙93; 95% confidence interval 0∙79 to 1∙10) compared to those in the program. The RR was attenuated when adjusted for risk factors; RR_adj_ = 0∙97 (0∙82 to 1∙15). The control group consisted of two subpopulations, those who only had a mammogram outside the program (RR_adj_ =1∙04; 0∙86 to 1∙26) and those who never had a mammogram (RR_adj_ = 0∙77; 0∙59 to 1∙01). These groups differed significantly with respect to risk factors for breast cancer, partly as a consequence of the prescription rules for hormone therapy which indicate a mammogram.

**Conclusions:**

In the fully implemented NBCSP, no significant difference was found in cumulative incidence rates of breast cancer between NOWAC women screened and not screened. Naïve comparisons of screened and unscreened women may be affected by important differences in risk factors. The current challenge for the screening program is to improve the diagnostics used at prevalence screenings (ages 50–51).

## Background

The public discussion following a large number of scientific articles related to overdiagnosis in national mammographic screening programs for breast cancer has become a major concern both for national screening programs and women deciding to participate. In the context of screening, overdiagnosis is the discovery of cancers that without screening would not have been diagnosed and consequently treated in a woman’s lifetime [1,2]. The main problem is the lack of diagnostic procedures that can subclassify breast tumors into overdiagnostic and clinically important invasive cancers which would obviate overtreatment. This limitation has forced researchers to try many different approaches to estimate the overdiagnosis [3-14]. An independent meta-analysis of three clinical trials reported a 19% increased incidence of breast cancer among screened women during the screening period and an 11% increased incidence if the years after the active screening were included [1]. Estimates based on ecological analyses are heterogeneous [6,8,9,11-13]. A major weakness of ecological analyses is the inability to adequately [15] control for the confounding effect of hormone therapy (HT). In Norway, as in most other countries, public guidelines for prescribing HT include an initial mammogram or participation in a national screening program [16,17]. Thus, the participants in the program will more often be users of HT. Since HT users have a higher risk of breast cancer [18,19], some of the estimated overdiagnosis might be due to the more extensive HT use among screening program participants. In addition, HT can reduce both mammogram sensitivity and specificity due to high breast density associated with HT use [20].

Estimates of overdiagnosis have included either only invasive cancers, or both invasive and ductal carcinoma *in situ* (DCIS) which are most often identified through mammography. Several recent cohort analyses were published from Norway [2], Denmark [21], and Italy [22] using a record linkage design with information on screening invitations or participations from program registries, and outcomes from cancer registries. The estimated overdiagnosis varied from almost zero to around ten percent when the years after the end of active screening were included. Individual level data were used to examine overdiagnosis in Norway, resulting in overdiagnosis estimates between 10 and 20 percent [2]. None of the studies had access to information on mammograms taken outside the program or necessary information for control of confounders or assessment of risk factors.

While the historical development of the screening program in Norway and many other countries has been used for estimating overdiagnosis, the core question for women entering the system today is the current and future level of risk of overdiagnosis in the national ongoing program. The Norwegian Breast Cancer Screening Program (NBCSP) has operated on a national scale since 2005 [23]. When estimating the consequence of participating in a mammographic screening program, three different screening phases are identified. First, prevalence screening occurs during the first participation. In the NBCSP, all women are first invited at 50 or 51 years of age. Later screening examinations (52–69 years), based on both the clinical and mammographic examinations compared to previous ones, give an incidence screening. Finally, the “compensatory drop” in the years after the age of 69 when the women are no longer offered screening. Since screening should detect cancers earlier than normally identified, there is expected to be a drop in the incidence when screening is stopped [10].

This analysis uses the national population based cohort Norwegian Women and Cancer study (NOWAC) to compare cumulative breast cancer incidence rates among women with different mammography histories between 52 and 79 years of age using incidence data for 2005–2010.

## Methods

### Norwegian Breast Cancer Screening Program (NBCSP)

The NBCSP started in 1996 as an evaluation project in four counties, but later expanded to the entire country [24]. It follows European guidelines with mammograms obtained in two views and each read independently by two radiologists. In 2005, the program was fully implemented in most of the country, but first invitations to the program were still being sent to all age groups (50–69) in two small counties: Hedmark and Vestfold. Starting in 2006, the screening program was fully implemented and all women were first invited to the program at 50 or 51 years. In 2006, there were 28 375 women with first invitations to the NBCSP, including 25 357 (89%) at ages 50–51, 1361 (5%) at ages 52–53 and the rest (6%) in older age groups (Cancer Registry of Norway, unpublished data). Women are then invited back every two years through age 69.

### Norwegian Women and Cancer (NOWAC)

NOWAC was initiated in 1991 [25]. Questionnaires were mailed to women randomly selected from the national population register held by Statistics Norway during 1991–2007. For each woman the unique person number, name, and address were extracted. Before mailing the letter of information and the questionnaire, the person number was replaced by a serial number that was the only identification on the questionnaire. The overall response rate for NOWAC questionnaires is 62%. All linkages between NOWAC members and national registries were done by Statistics Norway based on the unique person number.

The NOWAC questionnaires include information about mammography as well as lifestyle and social-demographics. During 2002–2005, the questionnaires included detailed questions about the type of mammogram. Women were asked if they had a mammogram and if so, how many were through an invitation to the NBCSP, through a referral from their doctor, or without an invitation or referral. Ninety-one percent of women aged 52 or older at the time of their submitted questionnaire answered these detailed mammography questions. Women who indicated that they had at least one examination via NBCSP invitation were considered as participating in the mammography program. During 2005–2007, after the nationwide implementation of the NBCSP, the referral questions were removed and instead women were asked how many years it had been since their last mammography examination. The change in questions was based on the assumption that information on participation could be taken from the screening register held by the Cancer Registry of Norway. However, this detailed information has not been available to researchers on an individual level, with a recent exception [2].

Although the questions about mammography were asked only once, the answers for women over age 52 were stable indicators of mammography patterns. A random subset of NOWAC participants who were asked about their mammography history in 2003 received another questionnaire during 2010–2011. For those who were 52 years or more at the time of the first questionnaire and answered a second questionnaire (N = 7361), 93% of those who reported never having had a mammogram on the first questionnaire had the same response. The answers regarding programmed mammograms were also robust. Of those indicating participation in the NBCSP, 83% responded the same 7 years later and for those indicating that they only had mammograms outside the NBCSP, 78% responded the same 7 years later.

### Sample selection

The NOWAC Cohort includes 172 478 women between the ages of 30 and 70 at recruitment who were randomly selected from the Norwegian population. A subset of these women were selected to form a Mammography Evaluation Cohort for this study. The sample was restricted to women who completed a NOWAC questionnaire during 2002–2007 at an age of 52 or higher and who lived in a county with a fully implemented screening program. These restrictions ensured that women in the study would have received at least one invitation to participate in the screening program prior to the questionnaire, thus determining which women were participating in the nation screening. The evaluation cohort also excluded women with a diagnosis of invasive breast cancer or DCIS prior to their questionnaire, and those who did not answer the mammography questions. The Mammography Evaluation Cohort includes 53 363 women divided into three groups based upon their mammography history. The “never had a mammogram” group includes all women who reported only “No” to the questions about mammogram history. Since the Mammography Evaluation Cohort necessarily precludes women in the prevalence screening (ages 50–51) in order to accurately identify those participating in the program, previously published rates for invasive breast cancer and DCIS for Norwegian women aged 50–51 were used for comparison [2].

### Person-years and follow-up

**Figure 1:**
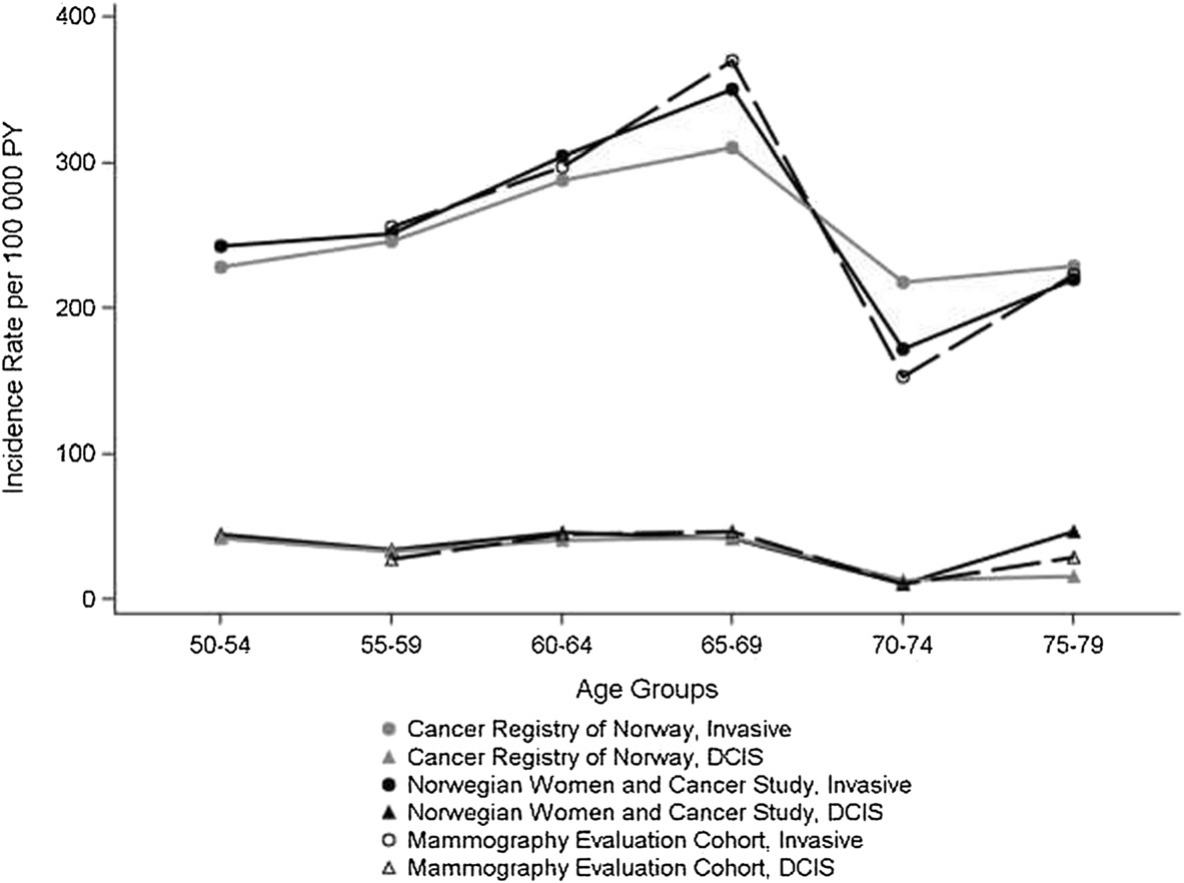
**Age-specific invasive and ductal carcinoma *in situ *(DCIS) breast cancer incidence rates 2006–2010.** Lines represent national figures, the Norwegian Women and Cancer Study and the Mammography Evaluation Cohort.

The number of people at risk for having a breast cancer diagnosis during 2005–2010 was calculated at the person level. Person-years (PY) were based on date of entrance into the study, age group, and date of exit from the study (date of invasive breast cancer diagnosis, death, or end of follow-up on 31 December 2010). Follow-up data during 2005–2010 came from the Cancer Registry of Norway and the Cause of Death Registry. Women in the Mammography Evaluation Cohort had an average follow-up time of 5∙6 years (median 6∙0) for a total of 300 016 PY, and 972 incident diagnoses of breast cancer (Table 1). The majority of these diagnoses were invasive breast cancer (89%) with the remaining DCIS (11%). The participation rate in the NBCSP for the cohort was 75%, which is similar to previously reported national participation rates of 76% [23]. As a randomly selected cohort, NOWAC participants have similar age-specific incidence rates as national figures [26] for 2006–2010 (Figure 1). NOWAC participants 65–69 years had a slightly higher incidence rate than those nationally, but the cumulative incidence rates were similar. The incidence rates for those in the Mammography Evaluation Cohort, the current study population, are representative of the overall cohort and thus comparable to national rates (dashed line in Figure 1; ages 55–79). For DCIS, the incidence rates were closely correlated.

**Table 1:** Age-specific breast cancer cases, person-years and rates for the Mammography Evaluation Cohort from the Norwegian Women and Cancer Study, 2005-2010

		**52-55**	**56-59**	**60-64**	**65-69**	**70-74**	**75-79**
**Total (N = 53363)**	Invasive	47	246	343	162	33	32
	Invasive + DCIS	53	267	400	181	35	36
	PY	22840	85593	114108	42796	20319	14360
	Rate	232	312	351	423	172	251
**Program (N = 42285)**	Invasive	35	206	284	130	26	19
	Invasive + DCIS	38	225	334	147	28	21
	PY	17674	70331	94926	33452	15045	8569
	Rate	215	320	352	439	186	245
**Outside program (N = 6479)**	Invasive	6	28	42	22	3	6
	Invasive + DCIS	8	30	49	24	3	8
	PY	2238	9244	12568	6069	3302	2724
	Rate	357	325	390	395	91	294
**Never (N = 4599)**	Invasive	6	12	17	10	4	7
	Invasive + DCIS	7	12	17	10	4	7
	PY	2929	6017	6615	3274	1972	3067
	Rate	239	199	257	305	203	228

DCIS = ductal carcinoma *in situ.*

PY = person-years.

Rates per 100,000 PY.

Program = received at least one mammogram within the screening program.

Outside program = received a mammogram, but only outside the screening program.

Never = never had a mammogram.

### Statistical analyses

Characteristics of the Mammography Evaluation Cohort groups were compared using chi-square tests of independence. Confidence intervals for the age-specific breast cancer incidence rates were calculated assuming a Poisson distribution [27]. Cumulative incidence rates for ages 52–79 were calculated as the cumulated sums of the age-specific incidence rates. Rates for each age were estimated from rates calculated for each age group assuming a constant rate within each group [28]. Log rank tests were used to compare cumulative incidence rates between groups. Age-adjusted relative risks and their 95% confidence intervals were estimated using Poisson regression with robust error variance [29].

The NOWAC questions before 2005 made it possible to perform the analyses taking into account three groups: *the program group* of women with at least one mammogram in the NBCSP*, the outside group* of women with mammograms only outside the screening program, and *the never group* of women who reported never having a mammogram. The last two groups were combined into *a “control” or reference group* for comparison with women participating in the screening program in order to be comparable with other analyses of program screened versus a control group. Estimates of relative risks were adjusted for major risk factors for breast cancer taken from the woman’s most recent questionnaire.

All statistical analyses were conducted in SAS version 9∙2 (SAS Incorporated, Cary, NC, USA). Statistical significance was defined as a two-sided test resulting in a p-value less than 0∙05.

## Results

The distribution of lifestyle factors related to breast cancer risk (Table 2) shows several distinct differences. Women who never had a mammogram tended to be older than those in the other groups, had more children, were less likely to have had a maternal history of breast cancer, and most distinctly, were less likely to be current users of HT (12% versus 25% for those in the program and 32% for those with mammograms outside the program). Differences between women in the control group, i.e., those who had a mammogram outside the program and those who never had a mammogram, were statistically significant for all risk factors (p < 0∙05).

**Table 2:** Characteristics of the Mammography Evaluation Cohort from the Norwegian Women and Cancer Study by mammogram history, 2005-2010

	Program*	**Outside program**	**Never**
N	42285	6479	4599
Age in 2005	N = 42276	N = 6476	N = 4440
52-55	23∙8%	19∙9%	25∙9%
56-59	36∙0%	31∙4%	24∙6%
60-64	26∙1%	24∙2%	19∙3%
65-69	7∙2%	11∙2%	8∙5%
70-74	5∙0%	6∙9%	8∙1%
75-79	2∙0%	6∙4%	13∙7%
Parity	N = 42285	N = 6479	N = 4599
none	7∙4%	7∙0%	8∙5%
1-2	52∙0%	52∙1%	43∙1%
3-4	36∙7%	36∙4%	40∙0%
5+	4∙0%	4∙5%	8∙4%
Age at first birth	N = 38904	N = 5983	N = 4170
<20	12∙0%	13∙3%	16∙4%
20-24	50∙6%	51∙1%	51∙6%
25-29	27∙9%	26∙9%	23∙3%
30-34	7∙1%	6∙4%	6∙3%
35+	2∙4%	2∙3%	2∙5%
Body mass index	N = 41054	N = 6288	N = 4369
<18.5	1∙1%	1∙0%	2∙2%
18.5-24,9	51∙4%	51∙4%	48∙8%
25-29,9	35∙2%	36∙5%	35∙1%
30+	12∙2%	11∙1%	13∙8%
Education	N = 40113	N = 6033	N = 4159
Primary (≤10)	38∙9%	41∙0%	51∙2%
Secondary (11–12)	29∙3%	29∙6%	23∙8%
College (≥13)	31∙8%	29∙4%	25∙0%
Maternal history of breast cancer	5%	8%	3%
Currently use hormone therapy	25%	32%	12%

*Program = received at least one mammogram within the screening program.

Outside program = received a mammogram, but only outside the screening program.

Never = never had a mammogram.

**Figure 2:**
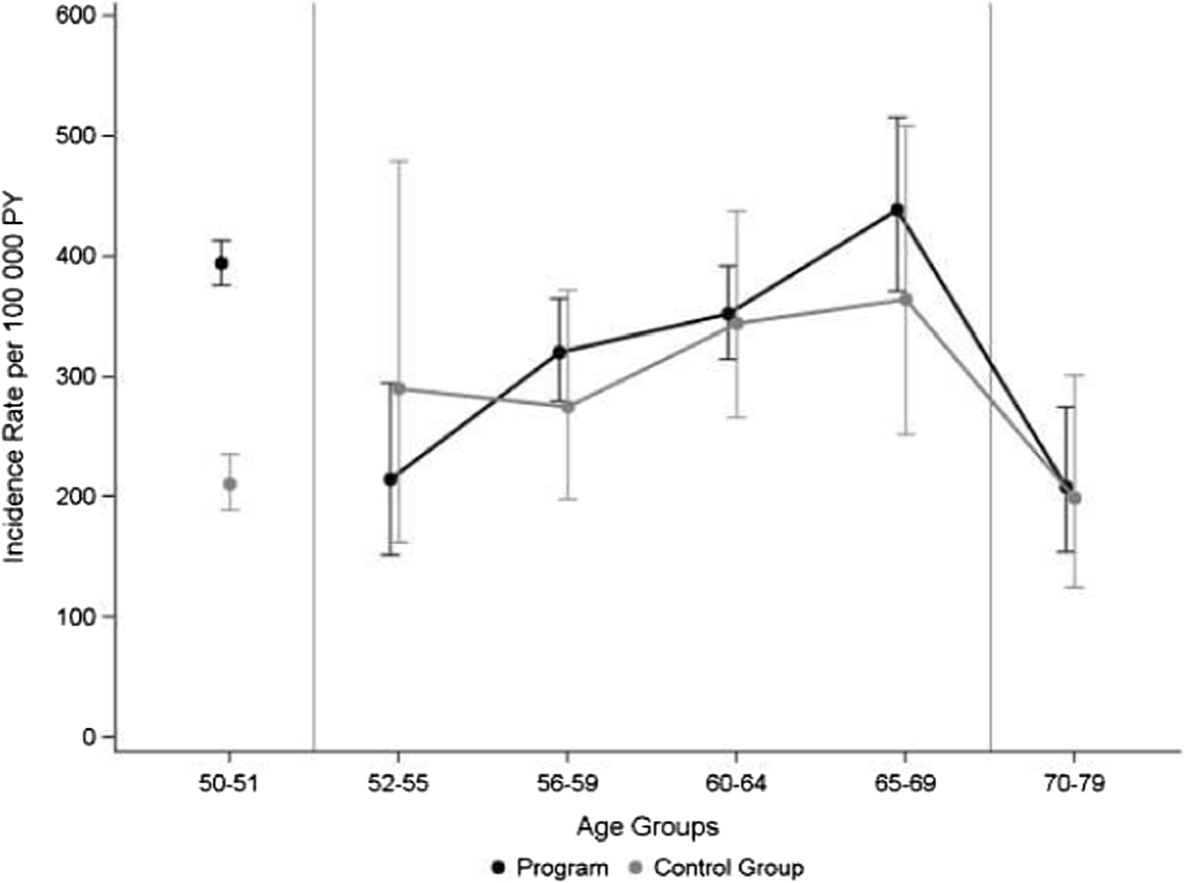
**Age-specific breast cancer rates and 95% confidence intervals by mammogram history.** Data for the Mammography Evaluation Cohort are from the Norwegian Women and Cancer Study, 2005–2010. The control group includes women who reported never having had a mammogram, and women who only had mammograms outside the screening program. Vertical lines separate prevalence screening (ages 50–51), incidence screening (ages 52–69), and follow-up (ages 70–79). The rates for ages 50–51 are from Falk et al. [2]. The last age category includes 10 years due to small numbers.

The age-specific incidence rates for the program group versus the control group showed similar rates across the study interval 52–79 years (Figure 2). Women who had a mammogram in the program and those who did not both demonstrated increasing rates of incident breast cancer during ages 52–69, although this was most evident in those who participated in the NBCSP. The drop in incidence rates for women over 69 years was clearly shown for both groups and was in the order of 200/100 000 PY from the preceding age group. Values for ages 50–51 are previously published rates of invasive breast cancer and DCIS for Norwegian women who attended the NBCSP during 1995–2009 (394/100 000 PY) and for those invited but who did not attend (211/100 000 PY) [2].

**Figure 3:**
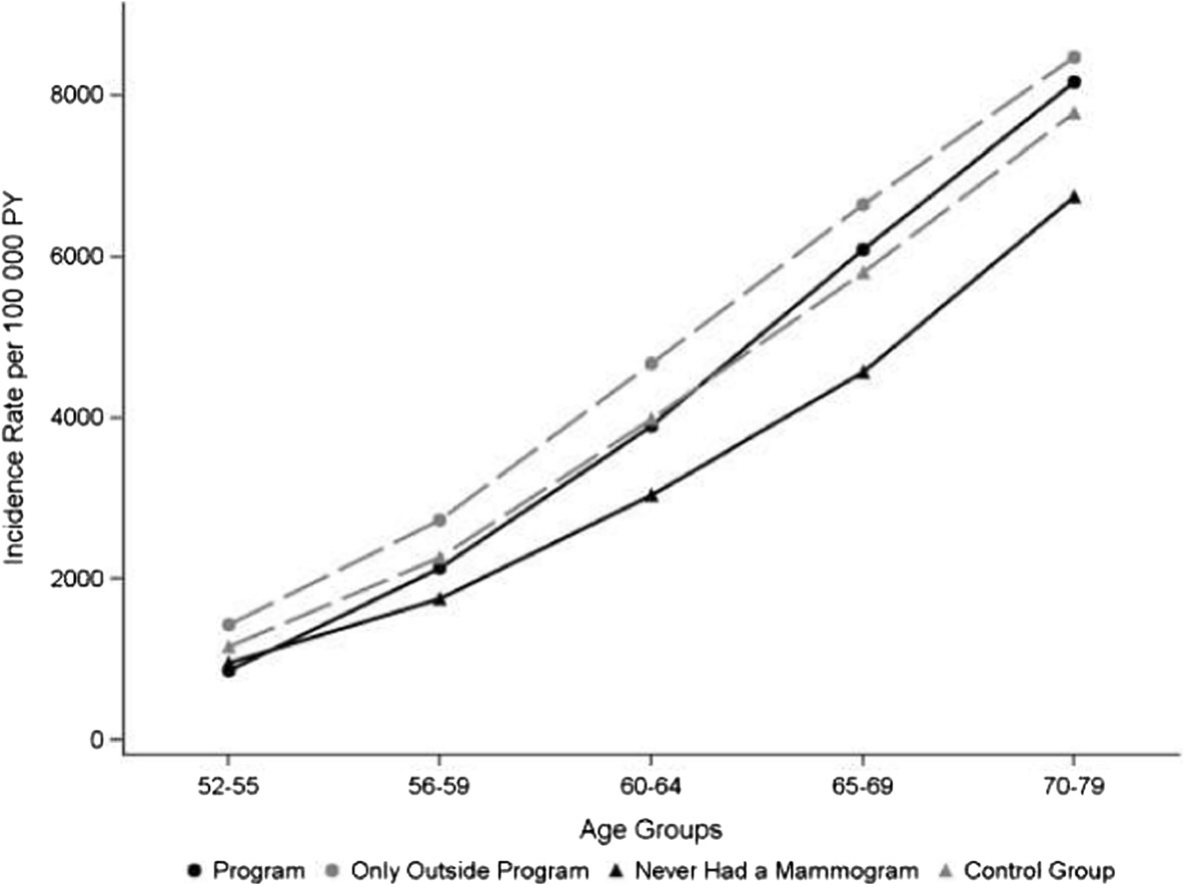
**Cumulative breast cancer rates by mammogram history.** Data for the Mammography Evaluation Cohort are from the Norwegian Women and Cancer Study, 2005–2010. The control group includes women who reported never having had a mammogram, and women who only had mammograms outside the screening program.

The cumulative incidence rates of invasive breast cancer and DCIS for women in the program and those in the control group were similar over the age-groups 52–79 years (Figure 3; p = 0∙47). The cumulative rate ratio for ages 52–79 was 1∙05 between the program and control groups. If the previously published rates for ages 50–51 are included, then the cumulative rate ratio for ages 50–79 is 1∙09 between the program and control groups. When examined separately, the heterogeneity of the control group becomes evident. Women who reported never having had a mammogram had the lowest cumulative rate of breast cancer, although it was not significantly different than those in the screening program (p = 0∙07). Women who reported only having had mammograms outside the screening program had the highest cumulative rate of breast cancer. Notably, the only significant difference in cumulative rates of cancer was between women who had mammograms outside the program and those who never had mammograms, i.e., between the two groups in the ‘control’ group (p = 0∙05). When cumulative rates of only invasive breast cancers are examined (not shown), the general patterns are the same although the differences in cumulated rates for ages 52–79 are much smaller among the groups.

The age-adjusted relative risks of having a breast cancer diagnosis for those who never had a mammogram compared with those in the program group showed a marginally-significant 23% reduced risk (RR = 0∙77; 95% CI: 0∙59 to 1∙01) (Table 3). The results were non-significant and attenuated after adjusting for important risk factors (RR_adj_ = 0∙82, 0∙61 to 1∙11). Women who had received a mammogram only outside the screening program had a slightly higher, although also non-significant, age-adjusted relative risk for a breast cancer diagnosis compared to those in the screening program (RR_adj_ = 1∙04, 0∙86 to 1∙26). The combined group of all women without a screening program mammogram (never had a mammogram or only outside screening) versus the program group showed an age-adjusted relative risk of 0∙93 (0∙79 to 1∙10). Further adjustments for risk factors attenuated the estimate; 0∙97 (0∙82 to 1∙15).

**Table 3:** Age-adjusted relative risk (RR) of breast cancer: estimates and 95% confidence intervals

**Invasive and DCIS**	**Never/outside program**	**Never**	**Outside program**
Age adjusted*	0∙93 (0∙79, 1∙10)	0∙77 (0∙59, 1∙01)	1∙04 (0∙86, 1∙26)
**Additionally adjusted for:**			
**Parity**	0∙94 (0∙80, 1∙10)	0∙78 (0∙59, 1∙02)	1∙04 (0∙86, 1∙26)
**Hormone therapy use**	0∙93 (0∙79, 1∙10)	0∙81 (0∙62, 1∙06)	1∙01 (0∙83, 1∙22)
**Maternal history of cancer**	0∙93 (0∙79, 1∙09)	0∙78 (0∙59, 1∙01)	1∙02 (0∙84, 1∙24)
**Body mass index (<25, 25+)**	0∙95 (0∙80, 1∙12)	0∙77 (0∙58, 1∙01)	1∙06 (0∙87, 1∙28)
**Education (primary, secondary, college)**	0∙96 (0∙81, 1∙14)	0∙77 (0∙58, 1∙03)	1∙07 (0∙88, 1∙30)
**Full model**	0∙97 (0∙82, 1∙15)	0∙82 (0∙61, 1∙11)	1∙05 (0∙86, 1∙28)
**Invasive only**	**Never/outside program**	**Never**	**Outside program**
Age adjusted*	0∙96 (0∙81, 1∙14)	0∙85 (0∙65, 1∙12)	1∙03 (0∙84, 1∙26)
**Additionally adjusted for:**			
**Parity**	0∙96 (0∙81, 1∙15)	0∙86 (0∙65, 1∙03)	1∙02 (0∙84, 1∙26)
**Hormone therapy use**	0∙96 (0∙81, 1∙14)	0∙90 (0∙69, 1∙18)	0∙99 (0∙81, 1∙22)
**Maternal history of cancer**	0∙96 (0∙81, 1∙13)	0∙86 (0∙65, 1∙12)	1∙01 (0∙83, 1∙25)
**Body mass index (<25, 25+)**	0∙97 (0∙82, 1∙16)	0∙85 (0∙64, 1∙12)	1∙05 (0∙85, 1∙29)
**Education (primary, secondary, college)**	0∙99 (0∙83, 1∙18)	0∙86 (0∙65, 1∙15)	1∙06 (0∙86, 1∙31)
**Full model**	1∙00 (0∙84, 1∙20)	0∙93 (0∙69, 1∙25)	1∙04 (0∙84, 1∙29)

Mammography Evaluation Cohort data are from the Norwegian Women and Cancer cohort, 2005–2010. Women in the mammogram screening program are the reference group. RR calculated for women who reported never having a mammogram (never) and those who only had mammograms outside the screening program (outside program).

*Age groups: 52–55, 56–59, 60–64, 65–69, 70–79.

## Discussion

This analysis did not show a significantly increased cumulative incidence rate in screened women versus other women from NOWAC in age-groups covering 52–79 years. Relative risks of those not screened compared with screened showed a 7% decreased risk, although non-significant. After adjusting for risk factors, the decreased risk was 3%. The control group, which did not participate in the screening program, included two quite different subpopulations: those who never had a mammogram, and those who only had them outside the program. These subpopulations differed significantly on risk factors and although the cumulative risk of breast cancer for those with mammograms outside the program was higher compared with those who never had a mammogram, the difference was non-significant.

The results and interpretations have some limitations. First, the cohort is small. This is clearly demonstrated in the analysis of different subgroups. Second, the information on screening participation was based on a self-report questionnaire of mammography history. It is unknown if some women in the control group may have had a screening program mammogram after their last questionnaire, although the small retest group suggests low bias. A source of systematic bias could be the introduction of digital technology between 2004 and 2011. This could give an additional prevalence screening in all age-groups dependent on the introduction in the different counties. The introduction was gradual and the last counties changed in 2011 (Cancer Registry of Norway, unpublished data).

On the other hand, NOWAC is one of very few national representative cohorts that can be used for the analysis of an ongoing screening program. The results clearly demonstrate the problem of comparing a control and a screened group and the interpretations of the control group concept. The control group in Norway consisted of women either with mammograms taken outside the program as a consequence of diagnostic procedures or opportunistic screening, and those without any mammograms. The relative structure of these groups might differ from country to country and over time, invalidating ecological analyses. The importance of the weights of the three groups was clearly related to the systematic differences in risk factors and by the underlying incidence rates. The most important was the confounding by current use of HT, which could be a serious bias in ecological analyses due to its known carcinogenic properties [30].

Another systematic selection bias between the two groups could be due to the Norway action plan for surveillance of breast cancer in carriers of certain BRCA mutations or evidence of risk of hereditary breast cancer without documented mutations [31]. This plan calls for annual mammograms for women from as young as 25 through age 60 and is independent of the NBCSP. Thus, these women are included in the group who had mammograms outside the NBCSP, and could explain the high prevalence of a maternal history of breast cancer of 8% in this group versus 3% among those who reported never having taken a mammogram. This differential prevalence of women with a maternal history of breast cancer could partially explain the differences in incidence.

The analysis included two age-related groups and background data on a third covering the ages of screening and a 10-year post-screening follow-up. The previously published data for the prevalence screening (ages 50–51) indicated that screened women had twice the rate of breast cancer during these ages as those who did not attend screening. Our analysis of the incidence screening (52–69 years) showed no significant differences according to mammogram history. There was a drop in breast cancer rates following the end of screening for both those screened and those not participating in the NBCSP, possibly related to repeated screenings outside the NBCSP for those in the control group.

The cumulative incident rates for the individual mammography histories elucidated the differences within the control group. Those who never had a mammogram had the lowest cumulative rate for ages 52–79, while those who only had mammograms outside the NBCSP had the highest cumulative rate. Although not significantly different, these subtle differences in cumulative rates mirror the significant differences in risk factors observed. These findings suggest that ecological comparisons among self-selected groups of mammography attendees may be misleading if they fail to account for the heterogeneity within the unscreened population.

Other approaches for the analysis of the Norwegian screening program used a cohort design with counties as proxy for individual information on screening participation [3]. This gave an overdiagnosis of 25% in the age-range 50–69 years or 15% including the “compensatory drop”. Again, the analysis was based on the assumption of a control group without knowledge of the use of mammography in that group. The Cancer Registry of Norway [2] recently estimated overdiagnosis in the order of 10-13% for invasive breast cancer and 14-20% for both DCIS and invasive breast cancers depending on if women were only invited to mammography or had multiple screening visits. The analysis was based on individual information on screening status in the national screening program, but did not examine risk factors. The discrepancies towards the estimates given based on the older clinical trials [1] could be due to the improvement in diagnostics over time. The estimate of overdiagnosis in the present analysis is lower than both with the UK independent estimate [1] and the estimate from the Cancer Registry of Norway [2], but did not include prevalence diagnoses. Given these published overdiagnosis estimates that included prevalence screenings, the high rates of breast cancer cases during ages 50–51, and our non-significant findings during ages 52–69, the data suggest that most overdiagnosis may occur during the prevalence screenings.

## Conclusions

The results from the present analysis differ from previous studies due to the focus on recent, modern screening in the context of the Norwegian health care system and with proper adjustment for important confounders. Future work on overdiagnosis should include examination of risk factors, especially use of HT, when providing estimates. The findings support that women participating in the ongoing, national mammographic screening program of breast cancer after its complete installment might only have overdiagnosis related to the prevalence screening. This should lead to a more careful diagnostic work-up for women during the initial prevalence screening and careful considerations of necessary treatment.

### Consent

All women have filled in an informed consent for later linkages to the Cancer Registry of Norway, the Norwegian Breast Cancer Screening Program, and the register of death certificates in Statistics Norway as well as the use of these data in research. The NOWAC study was approved by the Regional Committee for Medical and Health Research Ethics in North Norway. The Directorate of Health gave an exemption from the Norwegian rules of confidentiality for linking data using personal identifiers. Storage of data is in compliance with the rules of the Norwegian Data Inspectorate.

## Competing interests

The authors declare that they have no competing interests.

## Authors’ contributions

EL is the principle investigator of the Norwegian Women and Cancer study and contributed to all aspects of this study and manuscript preparation. NM contributed to the data analysis, data interpretation, and writing. MW and JCT contributed to the data interpretation and writing. All authors read and approved the final manuscript.

## Pre-publication history

The pre-publication history for this paper can be accessed here:

http://www.biomedcentral.com/1471-2407/13/614/prepub

## References

[B1] Marmot MG Altman DG Cameron DA Dewar JA Thompson SG Wilcox M Independent UKPBCS The benefits and harms of breast cancer screening: an independent review Lancet 2012 13 9855 1778 1786 23117178 10.1016/S0140-6736(12)61611-0

[B2] Falk RS Hofvind S Skaane P Haldorsen T Overdiagnosis among women attending a population-based mammography screening program Int J Cancer 2013 13 705 713 10.1002/ijc.28052 23355313 PMC3708102

[B3] Kalager M Adami HO Bretthauer M Tamimi RM Overdiagnosis of invasive breast cancer due to mammography screening: results from the norwegian screening program Ann Intern Med 2012 13 7 491 U461 10.7326/0003-4819-156-7-201204030-00005 22473436

[B4] Yen AMF Duffy SW Chen THH Chen LS Chiu SYH Fann JCY Wu WYY Su CW Smith RA Tabar L Long-term incidence of breast cancer by trial arm in one county of the Swedish Two-County Trial of mammographic screening Cancer 2012 13 23 5728 5732 10.1002/cncr.27580 22605639

[B5] Vannier MW Screening mammography: what good is it and how can we know if It works? J Natl Cancer Inst 2012 13 14 1039 1040 10.1093/jnci/djs289 22811440 PMC3731436

[B6] Gotzsche PC Nielsen M Screening for breast cancer with mammography Cochrane Database Syst Rev 2011 1 CD001877 21249649 10.1002/14651858.CD001877.pub4

[B7] Morrell S Barratt A Irwig L Howard K Biesheuvel C Armstrong B Estimates of overdiagnosis of invasive breast cancer associated with screening mammography Cancer Causes Control 2010 13 2 275 282 10.1007/s10552-009-9459-z 19894130

[B8] Jorgensen KJ Gotzsche PC Overdiagnosis in publicly organised mammography screening programmes: systematic review of incidence trends Br Med J 2009 13 b2587 10.1136/bmj.b2587 19589821 PMC2714679

[B9] Zahl PH Maehlen J Welch G The natural history of invasive breast cancers detected by screening mammography Arch Intern Med 2008 13 21 2311 2316 10.1001/archinte.168.21.2311 19029493

[B10] Biesheuvel C Barratt A Howard K Houssami N Irwig L Effects of study methods and biases on estimates of invasive breast cancer overdetection with mammography screening: a systematic review Lancet Oncol 2007 13 12 1129 1138 10.1016/S1470-2045(07)70380-7 18054882

[B11] Zackrisson S Andersson I Janzon L Manjer J Garne JP Rate of over-diagnosis of breast cancer 15 years after end of Malmo mammographic screening trial: follow-up study Br Med J 2006 13 7543 689 691 10.1136/bmj.38764.572569.7C 16517548 PMC1410836

[B12] Duffy SW Agbaje O Tabar L Vitak B Bjurstam N Bjorneld L Myles JP Warwick J Overdiagnosis and overtreatment of breast cancer - Estimates of overdiagnosis from two trials of mammographic screening for breast cancer Breast Cancer Res 2005 13 6 258 265 10.1186/bcr1354 16457701 PMC1410738

[B13] Zahl PH Strand H Maehlen J Incidence of breast cancer in Norway and Sweden during introduction of nationwide screening: prospective cohort study Br Med J 2004 13 7445 921 924 10.1136/bmj.38044.666157.63 15013948 PMC390204

[B14] Puliti D Duffy SW Miccinesi G de Koning H Lynge E Zappa M Paci E Grp EW Overdiagnosis in mammographic screening for breast cancer in Europe: a literature review J Med Screen 2012 13 42 56 22972810 10.1258/jms.2012.012082

[B15] Greenland S Morgenstern H Ecological bias, confounding, and effect modification Int J Epidemiol 1989 13 1 269 274 10.1093/ije/18.1.269 2656561

[B16] Norsk legemiddelhåndbok [Norwegian Medicine Handbook, In Norwegian] http://legemiddelhandboka.no/

[B17] Physicans Desk Reference [Felleskatalogen, In Norwegian] http://www.felleskatalogen.no/medisin/

[B18] Banks E Beral V Bull D Reeves G Austoker J English R Patnick J Peto R Vessey M Wallis M Breast cancer and hormone-replacement therapy in the Million Women Study Lancet 2003 13 9382 419 427 12927427 10.1016/s0140-6736(03)14065-2

[B19] Bakken L Alsaker E Eggen AE Lund E Hormone replacement therapy and incidence of hormone-dependent cancers in the Norwegian Women and Cancer study Int J Cancer 2004 13 1 130 134 10.1002/ijc.20389 15305384

[B20] Carney PA Miglioretti DL Yankaskas BC Kerlikowske K Rosenberg R Rutter CM Geller BM Abraham LA Taplin SH Dignan M Individual and combined effects of age, breast density, and hormone replacement therapy Use on the accuracy of screening mammography Ann Intern Med 2003 13 3 168 175 10.7326/0003-4819-138-3-200302040-00008 12558355

[B21] Njor SH Olsen AH Blichert-Toft M Schwartz W Vejborg I Lynge E Overdiagnosis in screening mammography in Denmark: population based cohort study BMJ 2013 13 f1064 10.1136/bmj.f1064 23444414 PMC3582341

[B22] Puliti D Zappa M Miccinesi G Falini P Crocetti E Paci E An estimate of overdiagnosis 15 years after the start of mammographic screening in Florence Eur J Cancer 2009 13 18 3166 3171 10.1016/j.ejca.2009.06.014 19879130

[B23] Cancer Registry of Norway Cancer in Norway 2009: Cancer incidence, mortality, survival and prevalence in Norway 2011 Oslo, Norway: Cancer Registry of Norway

[B24] Hofvind S Geller B Vacek P Thoresen S Skaane P Using the European guidelines to evaluate the Norwegian Breast Cancer Screening Program Eur J Epidemiol 2007 13 7 447 455 10.1007/s10654-007-9137-y 17594526

[B25] Lund E Dumeaux V Braaten T Hjartaker A Engeset D Skeie G Kumle M Cohort profile: The Norwegian women and cancer study - NOWAC - Kvinner og kreft Int J Epidemiol 2008 13 1 36 41 10.1093/ije/dym137 17644530

[B26] Cancer Registry of Norway Cancer in Norway 2010: Cancer incidence, mortality, survival and prevalence in Norway 2012 Oslo, Norway: Cancer Registry of Norway

[B27] Ulm K A simple method to calculate the confidence-interval of a standardized mortality ratio (SMR) Am J Epidemiol 1990 13 2 373 375 2296988 10.1093/oxfordjournals.aje.a115507

[B28] Boniol M Heanue M Curado MP, Edwards B, Shin HR, Storm J, Ferlay M, Heanue M, Boyle P Chapter 7: age-standardisation and denominators Cancer Incidence in Five Countinents 2007 IX Lyon France: International Agency for Research on Cancer

[B29] Zou G A modified poisson regression approach to prospective studies with binary data Am J Epidemiol 2004 13 7 702 706 10.1093/aje/kwh090 15033648

[B30] International Agency for Research on Cancer Combined estrogen-progestogen contraceptives and combined estrogen-progestogen menopausal therapy IARC Monographs on the Evaluation of Carcinogenic Risks to Humans. Volume 91 2007 Geneva, Switzerland: World Health Organization PMC4781221 18756632

[B31] National action plan with guidelines for diagnosis, treatment and follow-up of patients with breast cancer [In Norwegian] http://www.helsebiblioteket.no/retningslinjer/brystkreft/2-forebygging/2.2-oppf%C3%B8lging-av-kvinner

